# Expression of Pluripotency Genes in Chondrocyte-Like Cells Differentiated from Human Induced Pluripotent Stem Cells

**DOI:** 10.3390/ijms19020550

**Published:** 2018-02-12

**Authors:** Ewelina Stelcer, Katarzyna Kulcenty, Marcin Rucinski, Karol Jopek, Tomasz Trzeciak, Magdalena Richter, Joanna P. Wroblewska, Wiktoria M. Suchorska

**Affiliations:** 1 Radiobiology Lab, Greater Poland Cancer Centre, Garbary 15th Street, 61-866 Poznan, Poland; katarzyna.kulcenty@wco.pl (K.K.); wiktoria.suchorska@wco.pl (W.M.S.); 2 The Postgraduate School of Molecular Medicine, Medical University of Warsaw, Zwirki i Wigury 61 Street, 02-091 Warsaw, Poland; 3 Department of Electroradiology, Poznan University of Medical Sciences, Garbary 15th Street, 61-866 Poznan, Poland; 4 Department of Histology and Embryology, Poznan University of Medical Sciences, Swiecickiego 6 Street, 60-781 Poznan, Poland; marcinruc@ump.edu.pl (M.R.); kjopek@ump.edu.pl (K.J.); 5 Department of Orthopedics and Traumatology, Poznan University of Medical Sciences, 18 czerwca 1956r Street, 61-545 Poznan, Poland; trzeciak@orsk.ump.edu.pl (T.T.); mrichter@ump.edu.pl (M.R.); 6 Department of Pathology, Poznan University of Medical Sciences, Greater Poland Cancer Centre, Garbary 15th Street, 61-866 Poznan, Poland; joanna.wroblewska@wco.pl

**Keywords:** human induced pluripotent stem cells, chondrogenic differentiation, gene expression profile

## Abstract

Human induced pluripotent stem cells (hiPSCs) constitute an important breakthrough in regenerative medicine, particularly in orthopedics, where more effective treatments are urgently needed. Despite the promise of hiPSCs only limited data on in vitro chondrogenic differentiation of hiPSCs are available. Therefore, we compared the gene expression profile of pluripotent genes in hiPSC-derived chondrocytes (ChiPS) to that of an hiPSC cell line created by our group (GPCCi001-A). The results are shown on heatmaps and plots and confirmed by Reverse Transcription Quantitative Polymerase Chain Reaction (RT-qPCR) analysis. Unlike the ChiPS, our GPCCi001-A cells maintained their pluripotency state during long-term culture, thus demonstrating that this cell line was comprised of stable, fully pluripotent hiPSCs. Moreover, these chondrocyte-like cells not only presented features that are characteristic of chondrocytes, but they also lost their pluripotency, which is an important advantage in favor of using this cell line in future clinical studies.

## 1. Introduction

Stem cells (SCs) are a highly promising approach to tissue engineering. These cells could be used to treat a wide range of orthopedic disorders (such as articular cartilage defects) in which the capacity for self-repair is limited due to the lack of blood vessels, lymphatic vessels, and nerve cells [1]. The use of SCs may overcome the numerous drawbacks of autologous chondrocytes: the limited number of chondrocytes isolated for cell culture, difficulty in preserving their chondrogenic potential, and the re-differentiation of cells during tissue formation after implantation [2]. Defined transcription factors can be used to transform terminally-differentiated cells into human-induced pluripotent stem cells (hiPSCs), giving rise to SCs that share a wide range of characteristics with human embryonic SCs (hESCs) [3]. Thus, hiPSCs differentiated into chondrocytes may represent a novel stem cell-based approach to treat cartilage damage in the head and neck region and articular cartilage. The advantages of hiPSCs are numerous, including unlimited self-renewal capacity, high developmental plasticity, and reduced immunogenic properties [4]. In our laboratory, we obtained a hiPSC cell line derived from human primary dermal fibroblasts. This feeder-dependent hiPSC line (GPCCi001-A) was generated by using a modified EF1a-hSTEMCCA-loxP, a polycistronic vector composed of four reprogramming factors (OCT4, SOX2, KLF4, c-MYC) and a regulatory element (tetracycline-operator: stem cell cassette STEMCCA-tetO) [5]. Numerous studies have described the main techniques to perform chondrogenic differentiation in hiPSCs, the most common being the formation of embryoid bodies (EBs) [6,7]. Other methods include micromass culture [8], pellet culture [9], and directed differentiation [10]. Efficient in vitro chondrogenesis requires adding exogenous defined growth factors (GFs) from the transforming growth factor β superfamily to a chondrogenic medium. However, most of the aforementioned chondrogenesis methods require numerous steps that substantially lengthen the time needed to complete the in vitro differentiation process. Importantly, our understanding of certain key aspects of the cell differentiation process is incomplete, including how specific chondrogenic processes affect the gene expression profile of chondrocyte-like cells [11].

Recently, our group [12] developed a novel chondrogenic differentiation protocol. In this protocol, a monolayer is cultured for 3 weeks using a relatively small amount of mesodermal and chondrogenic GFs. The advantage of this directed differentiation protocol is that, unlike most other differentiation techniques (such as EB formation), our protocol requires no additional, time-consuming steps; as a result, differentiation can be achieved in only 21 days, which is a notable time and cost advantage over other protocols. Moreover, as we reported previously, these cells demonstrate desirable features that are characteristic of mature chondrocytes [12].

In this context, we carried out the current study—in which we performed a global gene expression analysis using the Affymetrix platform—to better understand the processes directing cell fate. This study had two primary aims: (1) to evaluate the similarities between hiPSC-derived chondrocytes (ChiPS) and “parental” hiPSCs at the gene level, (2) to assess how the differentiation process changes the expression of essential genes involved in pluripotency; and additional goal to determine whether the hiPSC cells maintain their pluripotency state in long term cultures.

Our findings suggest that differentiated cells lose their pluripotent state. The key genes involved in maintaining the self-renewal and pluripotency pathways are significantly down-regulated, a finding that also suggests a lower risk of potentially uncontrolled proliferation and, consequently, a reduced risk of undesirable tumorigenesis. We also confirmed that the hiPSCs obtained in our laboratory maintained their pluripotency state. We successfully obtained stable pluripotent SCs with the capacity to differentiate into the derivatives of three primary germ layers.

This work contributes to a better understanding of the processes and mechanisms responsible for diminishing pluripotency during in vitro chondrogenic differentiation. These data may be useful to evaluate the real potential and safety of the differentiation protocol developed by our group.

## 2. Results

### 2.1. Derivation of hiPSCs and Directed Differentiation into Chondrocytes

**Figure 1:**
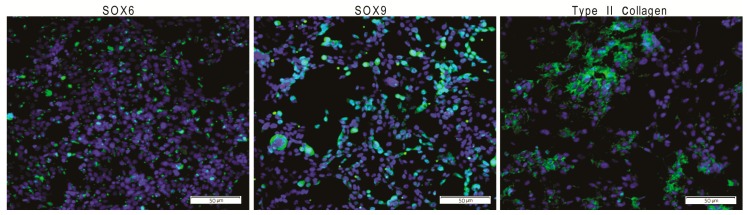
The GPCCi001-A cell line has the capacity to successfully differentiate into chondrogenic lineage (ChiPS) via previously established protocols. ChiPS cells reveal the presence of desirable chondrogenic markers, including SOX6, SOX9, and type II collagen visible as a green fluorescence. Nuclei are counterstained with DAPI.

All experiments used hiPSCs generated from human dermal primary fibroblasts according to a previously established protocol [5]. To generate the hiPSC line (GPCCi001-A) a lentiviral transfection involving modified EF1a-hSTEMCCA-loxP with tetO operator was applied [5]. Next, hiPSCs were differentiated into chondrocyte-like cells via monolayer culture (DIRECT differentiation) with the addition of the following defined GFs: FGF-2, BMP-4, PDGF, TGF-β3, and IGF-1 [12] (Figure 1).

These differentiated cells presented features that were characteristic of chondrocytes. Finally, global gene expression and bioinformatics analyses were carried out.

### 2.2. Analysis of Microarray Experiments of Gene Expression Profiling: In Contrast to Parental hiPSCs, hiPSC-Derived Chondrocytes (DIRECT) Do Not Demonstrate Pluripotency and Self-Renewal

**Figure 2:**
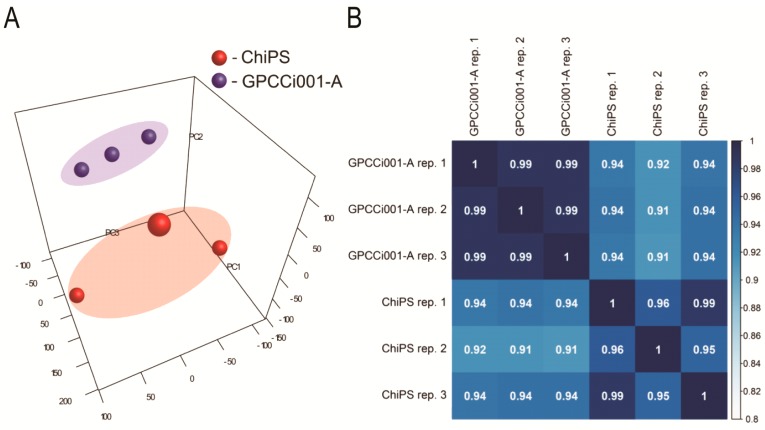
Microarray data analysis. (**A**) Principal Component Analysis (PCA) plot shows two distinct clusters: human induced pluripotent stem cells (hiPSCs) (GPCCi001-A cell line) (blue bubbles located into the blue area) and ChiPS generated from hiPSCs via chondrogenic differentiation in vitro (red bubbles located into the red area). To further investigate the expression differences/similarities between investigated cells (ChiPS vs. GPCCi001-A) Pearson’s correlation coefficient analysis of datasets (PC1—Principal Component 1; PC2—Principal Component 2; PC3—Principal Component 3) (**B**) was performed.

To perform a complete comparison of ChiPS and GPCCi001-A transcriptome profiles, we analyzed whole gene expression using Affymetrix Human Gene 2.1 ST ArrayStrips. These strips were used to examine the expression of 40,716 different transcripts. Principal component analysis (PCA) (Figure 2A) indicate a clear segregation of GPCCi001-A and ChiPS cells. The correlation coefficient analysis demonstrated that the expression differences at the whole transcriptome level are different between ChiPS and GPCCi001-A cells (Figure 2B).

**Figure 3:**
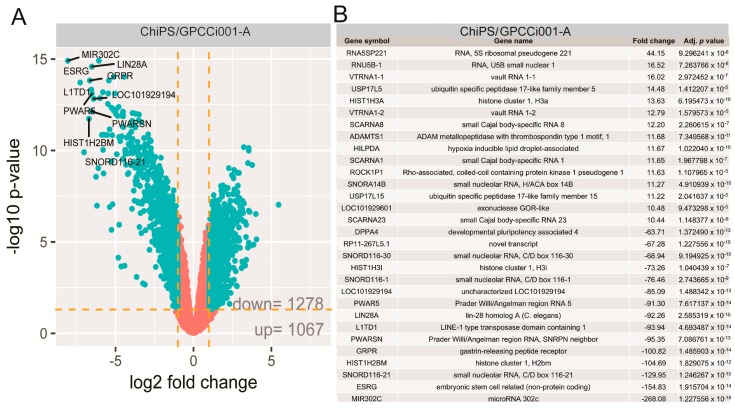
Volcano plots show the total gene expression profiles of the ChiPS and GPCCi001-A experimental groups. Each dot represents the mean expression level (*n* = 3) of a single gene obtained from a microarray normalized dataset. The orange dotted lines (cut off values) were established according to the following parameters: fold > |2| and adjusted *p*-value < 0.05. Genes above the cut-off are considered to be differentially expressed and are shown as blue dots. The total number of differentially expressed genes are displayed in the bottom right corner of the graph. The top ten most up- and down-regulated genes are described by their gene symbols (**A**). The table shows the 30 genes with the highest (15 genes) and lowest (15 genes) fold changes obtained from the list of differentially expressed genes (**B**).

The general profile of whole gene expression in the ChiPS and GPCCi001-A groups is shown in a volcano plot (Figure 3A), with each dot on the graph corresponding to one transcript.

The selection criteria of a significantly changed gene expression was based on an expression fold difference > absorbance (abs.) 2 and an adjusted *p*-value ≤ 0.05. Based on these criteria, 1278 genes were significantly down-regulated and 1067 genes were up-regulated in the ChiPS group versus the GPCCi001-A cells. The 15 genes with the highest and lowest fold change values are presented in tabular format displaying the gene symbol, gene name, fold change, and adjusted *p*-value (Figure 3B).

These genes were characterized by extremely high fold change values, especially for downregulated genes (range for upregulated genes: 44.15–10.44, and for downregulated genes: −268.08–−63.71). Some of these genes belonged to a group of genes directly involved in pluripotency and differentiation (*LIN28A*, *L1TD1*), while others belong to small nucleolar RNAs (*SNORD116-21*, *SNORD116-1, SNORD116-30, SCARNA23, SNORA14B, SCARNA1, SCARNA8*), or long non-coding RNA (*PWAR5, ESRG, PWARSN, RP11-267L5.1*) or are implicated in epigenetic regulation (*HIST1H3A, HIST1H2BM, HIST1H3I*).

**Figure 4:**
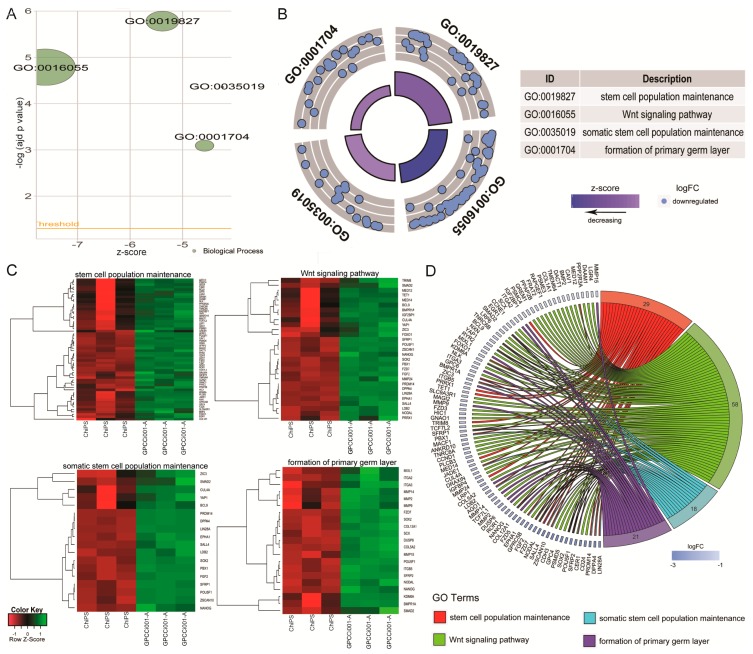
Bubble plot of four significantly-enriched Gene Ontology (GO) terms related to pluripotency regulation and differentiation, which were: “stem cell population maintenance”, “Wnt signalling pathway”, “somatic stem cell population maintenance”, and “formation of primary germ layer”. The negative logarithm of the adjusted *p*-value from all analyzed GO terms is given on the *y*-axis, while the *Z*-score value is shown on the *x*-axis (**A**). The circular scatter plot shows the differentially expressed genes assigned to each GO term. The logarithm of the fold change value (logFC) of differentially expressed genes is shown (**B**). Heat map graphs of genes from the following GO terms: “somatic stem cell population maintenance”, “stem cell population maintenance”, “formation of primary germ layer”, “Wnt signalling pathway”. Arbitrary signal intensity acquired from the microarray analysis is represented by the colours (green—higher; red—lower expression). Log2 signal intensity values for any single gene were resized to Row *Z*-Score scale. Genes belonging to the relevant GO term are described by their symbols (**C**). Circos plot demonstrating relationship between selected GO terms and their genes. Genes are located on the left side of the graph and indicated by their symbols. Genes were ordered based on their logFC values. Gene involvement in the GO terms was determined by connecting lines (**D**).

The most notable increase was observed in the RNA expression of 5S ribosomal pseudogene 221; RNA, U5B small nuclear 1, and the vault RNA 1-1 genes, whose respective fold change values were 44.15, 16.52, and 16.02. The most downregulated genes were microRNA302c (−268.08), embryonic stem cell related (non-protein coding; −154.83) and small nucleolar RNA, C/D box 116-21 (−129.95) (*p* < 0.05). Differentially expressed gene set was assigned to significantly enriched Gene Ontology (GO) terms related to the regulation of pluripotency and differentiation. This analysis, based on statistically significant GO terms, concerned the following biological processes: “stem cell population maintenance” (adjusted *p*-value = 1.65 × 10^−6^), “Wingless-type MMTV integration site family (Wnt) signalling pathway” (adjusted *p*-value = 1.63 × 10^−5^), “somatic stem cell population maintenance” (adjusted *p*-value = 4.37 × 10^−5^), “formation of primary germ layer” (adjusted *p*-value = 8 × 10^-4^). These results were visualized using the GOplot library [13]. The general characteristic of enriched GO terms is presented as a bubble plot where the negative logarithm of the adjusted *p*-value is assigned to the *y*-axis, with the *Z*-score value shown on the *x*-axis. The *Z*-score was calculated automatically using the GOplot library; the *Z*-score value indicates whether the process decreased (negative value) or increased (positive value). Gene sets assigned to selected ontological groups showed a significant decrease in gene expression in the ChiPS group, as reflected by negative *Z*-scores (Figure 4A).

The logarithm of the fold change (logFC) of differentially expressed genes assigned to each GO term is shown in Figure 4B, where each blue circle displays the down-regulated genes assigned to each of the GO terms. Differentially expressed genes assigned to each GO term were also subjected to a hierarchical clusterization algorithm and presented as heatmaps. Arbitrary signal intensities from selected genes are represented by colors (green = higher expression and red = lower expression). Log2 signal intensity values for any single gene were resized to row *Z*-Score scale. Additionally gene symbols are also shown (Figure 4C).

Of the differentially expressed genes, 29 were assigned to “stem cell population maintenance”, 58 to “Wnt signaling pathway”, 18 to “somatic stem cell population maintenance”, and 21 to “formation of primary germ layer”. Because of the gene ontology database structure, single genes can often be assigned to many ontological terms. For this reason, the relationship between genes and GO terms was mapped with a circos plot with visualization of logFC and gene symbols (Figure 4D).

**Figure 5:**
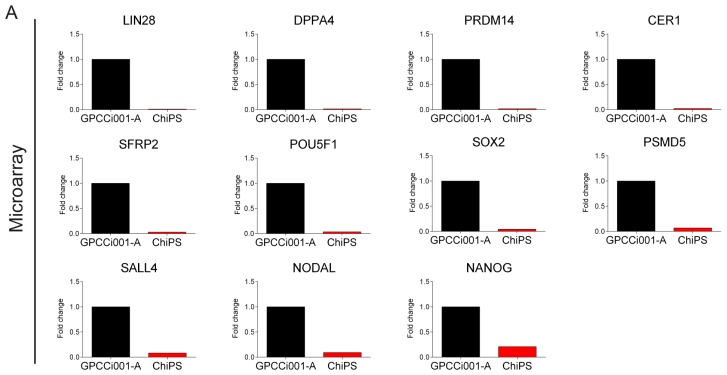

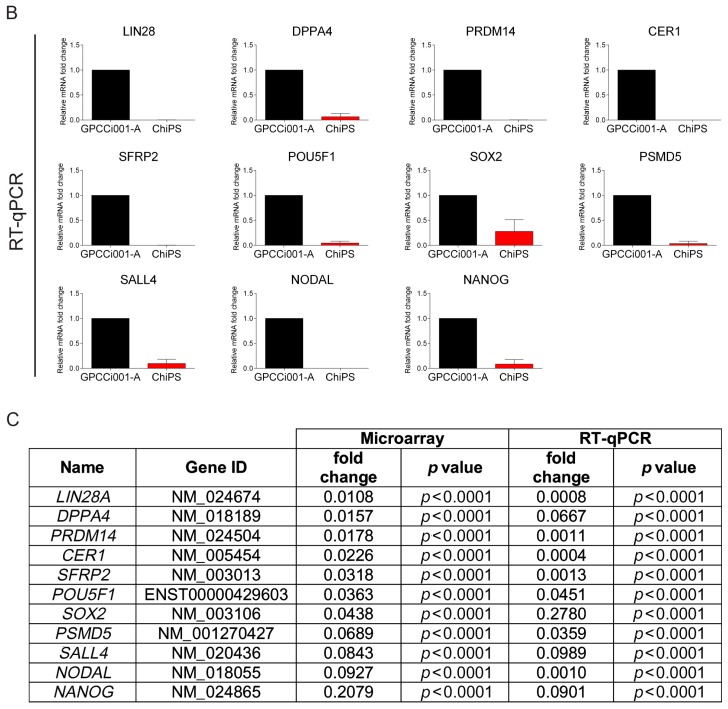
Real time qPCR validation of microarray data. For validation, we selected the most highly expressed genes according to the Circos plot GO terms. The top panel represents normalized GPCCi001-A cell line fold changes of selected genes based on microarray data (**A**). The bottom panel represents microarray data validated by RT-qPCR. The graph represents means ± SD from three independent experiments (**B**). The table representing normalized to GPCCi001-A fold changes and *p*-values of selected genes from microarray and RT-qPCR analysis (**C**).

All of the genes were down-regulated in the ChiPS group. The circos plot shows the genes that are the most downregulated in ChiPS versus GPCCi001-A cells. Based on this analysis, we selected the most downregulated genes (*LIN28A*, *DPPA4*, *PRDM14*, *CER1*, *SFRP2*, *POU5F1*, *SOX2*, *PSMD5*, *SALL4*, *NODAL*, and *NANOG*) for further qPCR evaluation. All of these genes are engaged in several different hiPSC pathways inter alia Wnt signalling and SC population maintenance pathways (Figure 5).

The accuracy of this study was verified by a detailed gene expression analysis (RT-qPCR) (Figure 5). In contrast to GPCCi001-A cells, ChiPS cells express genes involved in pluripotency and self-renewal at a very low level. ChiPS cells also present the downregulated expression of genes involved in the formation of primary germ layers. By contrast, the GPCCi001-A cells demonstrated a high expression of all of the evaluated genes.

## 3. Discussion

We have previously established protocols to successfully reprogram human fibroblasts into hiPSCs and to further differentiate these hiPSCs into chondrocyte-like cells (Figure 1 and Figure S1). However, before such cells can be used clinically, it is essential to demonstrate that they not only possess desirable pluripotency features but also that they maintain these features during long-term culture. The present study had two main aims: (1) to confirm that the chondrocyte-like cells (i.e., the ChiPS) obtained through our differentiation protocol lost their pluripotency nature and (2) to demonstrate that the GPCCi001-A cell line created by our group maintained self-renewal and pluripotency characteristics during long-term culture. Our findings showed that the chondrocyte-like cells do not express genes responsible for maintaining the pluripotency state whereas the GPCCi001-A cell line has the capacity to effectively differentiate into the derivatives of three primary germ layers. Below, we discuss our results in the context of more recent data on gene expression profile of hiPSCs and their derivatives differentiated in vitro. Most of the published data in this regard concerns research into the development of the nervous system and nervous-system disorders. In this sense, our research involving chondrogenesis constitutes a novel approach to investigating thoroughly the gene expression profile of obtained chondrocyte-like cells from stable hiPSCs (Figure S2).

According to Trevisan et al. [14] method of reprogramming—despite the different reprogramming efficiency—does not influence the gene expression profile of obtained hiPSCs. Those authors generated hiPSCs from fibroblasts via retroviral, Sendai virus and episomal vectors reprogramming and compared key features involved in stemness and pluripotency of obtained hiPS cell lines. They concluded that three investigated method of reprogramming give rise to equivalent hiPSCs with capacity to form EBs and similar expression profiles [14].

The available evidence [15] indicates that hiPSCs derived from individuals with Timothy syndrome can be further differentiated into neuronal precursors and neurons. However, the gene expression profile of those differentiated cells points to the presence of calcium signalling and abnormalities that emerge during the differentiation process. Those cells are characterized by alterations in the expression of genes present in lower cortical layers and callosal projection neurons, and by involvement in the tyrosine hydroxylase activity, and in the production of norepinephrine and dopamine [15].

Using next generation sequencing (RNA-Seq), [16] found that hiPSCs reprogrammed from fibroblasts by retroviral long terminal repeat (LTR) show dramatic changes in the splice isoform generation and in the expression of long non-coding RNAs such as HOTAIRM1—a cis-acting regulator of the HOXA cluster during neuronal differentiation in vitro [16]. Our results confirm this finding, particularly the strong changes that we observed in small and long noncoding RNAs (e.g., SNORD116-21, SNORD116-1, and RNU5B-1) during chondrogenic differentiation.

Another group [17] established an hiPSC cell line from fibroblasts isolated from a patient with relapsing-remitting multiple sclerosis (MS) by retroviral transduction using OCT4, SOX2, KLF4 and c-MYC factors (MSiPS). They then differentiated the MSiPS into astrocytes, oligodendrocytes, and neurons. Global analyses showed that the gene expression of MSiPS and hESCs (H9 cell line) presented similar levels of pluripotency genes (OCT 3/4, REX1 and SOX2) and demethylation compared to human dermal fibroblasts [17].

Linta and co-workers [18] investigated the gene expression profile of ion channels of keratinocyte-cell derived hiPSCs and keratinocytes, finding that the hiPSCs were characterized by specific an ion channel expression pattern. Additionally, the hiPSCs expressed a much higher number of ion channels compared to keratinocytes. Moreover, their differentiated progenitors (such as neurons and cardiomyocytes) also had a gene expression profile of various channel families and their subtypes that significantly differed from that of parental hiPSCs [18].

Mallon and co-workers [19] created an hiPSC cell line from neural precursor cells (NPC) obtained by differentiating H1 (WA01) hESCs using the STEMCCA kit. After differentiation, they then compared the gene expression and methylation profile of all three lines (hiPSCs, hESCs, NPCs), finding that the two PSC cell lines possessed comparable gene expression and methylation profiles, in contrast to the NPCs. The most notable difference was that hiPSCs were more prone to down-regulation due to increased methylation [19].

Despite the numerous studies described above, until now there has been a notable lack of data on the gene expression profile of chondrocyte-like cells differentiated from hiPSCs. To address this lack of data, we conducted the study presented here, in which we compared the gene expression profile of hiPSCs and ChiPS which were created in our laboratory, with a particular focus on the most important biological process involved in pluripotency and differentiation.

## 4. Materials and Methods

### 4.1. Chondrogenic Differentiation of hiPSCs

GPCCi001-A cells were cultured for one week in a pro-mesodermal medium. Then the medium was replaced with a standard chondrogenic medium supplemented with TGF-β3 (10 ng/mL) (ImmunoTools, Friesoythe, Germany) for another week. Next, that medium was replaced with a chondrogenic medium supplemented with TGF-β3 (10 ng/mL) and IGF-1 (10 ng/mL) (Peprotech, London, UK) for one final week of enhanced chondrogenesis in vitro according to a previously established protocol [12].

### 4.2. Immunofluorescence Analysis

The cells were transferred into a 0.1% gelatin-coated 48-well plate for 48 h and then washed with phosphate buffered saline (PBS) (Sigma Aldrich, Saint Louis, MO, USA) and fixed for 20 min in 100% methanol (SOX6 and SOX9) (CHEMPUR, Piekary Slaskie, Poland) or 4% formaldehyde (type II collagen) (CHEMPUR, Piekary Slaskie, Poland) (400 μL of methanol/formaldehyde per well). Then, the cells were rinsed with PBS containing 1% bovine serum albumin (BSA) (Sigma Aldrich, Saint Louis, MO, USA) and incubated for 30 min in PBS containing 1% BSA and 0.2% Triton X-100 (Sigma Aldrich, Saint Louis, MO, USA). After 30 min, the cells were washed with PBS containing 1% BSA. The primary antibodies were diluted in PBS containing 1% BSA and 0.2% Triton X-100 and the cells were incubated overnight at 4 °C with the following primary antibodies (all from Abcam PLC, Cambridge, UK): type II collagen (1:100) (ab34712); SOX6 (1:50) (ab30455); SOX9 (1:50) (ab59252). After conjugation with the primary antibodies, the cells were rinsed three times with PBS containing 1% BSA. The following secondary antibody was diluted with 1% BSA in PBS and incubated in the dark for 1 h at 37 °C: rabbit polyclonal antibody (1:500) (711-546-152) (Jackson ImmunoResearch, Philadelphia, PA, USA). After the cells were washed three times with 1% BSA in PBS, they were stained for 5 min with diamidino-2-phenylindole dye (DAPI) (Sigma Aldrich, Saint Louis, MO, USA) solution in water (1:10,000) and then washed with PBS before undergoing microscope analysis.

### 4.3. RNA Extraction

Total RNA isolation was carried out using the modified Chomczynski method with the TRI Reagent (Sigma, Saint Louis, MO, USA) and RNeasy Mini Elute cleanup Kit (Qiagen, Hilden, Germany), according to the manufacturer’s guidelines. The concentration of the total RNA was determined spectrophotometrically by measuring absorbance at 260 nm. The purity of the isolated RNA was determined by using the 260/280 nm absorption ratio, which was >1.8, as assumed (NanoDrop spectrophotometer, Thermo Scientific, Waltham, MA, USA). The quality and integrity of RNA was also checked in a Bioanalyzer 2100 (Agilent Technologies, Inc., Santa Clara, CA, USA). The resulting RNA integrity numbers (RINs) ranged from 8.5 to 10, with a mean of 9.2 (Agilent Technologies, Inc., Santa Clara, CA, USA). Each RNA sample was diluted to a concentration of 50 ng/μL. For the microarray experiments, 100 ng of total RNA was used. The remaining isolated RNA material was used for the RT-qPCR study.

### 4.4. Microarray Expression Studies

The microarray study was conducted according to the detailed procedures described elsewhere [13,20,21,22]. The entire procedure for preparing RNA for hybridization was performed using the GeneChip Whole Transcript (WT) PLUS Reagent Kit (Affymetrix, Santa Clara, CA, USA). One-hundred ng of RNA was subjected to a two-step cDNA synthesis reaction using random primers extended by the T7 RNA polymerase promoter sequence. The synthesis of complementary RNA (cRNA) was performed by using in vitro transcription (16 h, 40 °C). cRNA was purified and re-transcribed into cDNA, which was biotin labeled and fragmented using the Affymetrix GeneChip WT Terminal Labeling and Hybridization kit (Affymetrix, Santa Clara, CA, USA). Biotin-labeled fragments of cDNA (5.5 μg) were hybridized by the Affymetrix Human Gene 2.1 ST ArrayStrip (20 h, 48 °C). After the hybridization, the microarrays were subjected to a washing and staining procedure, performed according to the technical protocol, using the Affymetrix GeneAtlas Fluidics Station (Affymetrix, Santa Clara, CA, USA). The array strips were scanned using an Imaging Station of GeneAtlas System (ThermoFisher Scientific, Waltham, MA, USA). The preliminary analysis of the scanned chips was performed with the Affymetrix GeneAtlas Operating Software (Affymetrix, Santa Clara, CA, USA). The quality of gene expression data was checked using the quality control criteria provided by the software.

### 4.5. Microarray Data Analysis

The obtained cell intensity (CEL) files were imported for further analysis using the R statistical language and Bioconductor package with the relevant Bioconductor libraries (www.bioconductor.org). The Robust Multiarray Average (RMA) normalization algorithm implemented in the “Affy” library (https://www.bioconductor.org/packages/release/bioc/html/affy.html) was used for normalization, background correction, and calculation of the expression values of all of the examined genes [23]. Exploratory data evaluation by Principal Component Analysis (PCA) and correlation coefficient analysis was carried out to individuate distances/similarities and gradients/patterns in the gene expression profile among the analyzed hiPSCs and ChiPS cells (three replicates per group). Biological annotations were taken from “pd.hugene.2.1.st” (http://bioconductor.org/packages/release/data/annotation/html/pd.hugene.2.1.st.html). This library was used for the mapping of normalized gene expression values with their symbols, gene names and Entrez IDs, leading to a complete gene data table. Differential expression and statistical assessment were determined by using the linear models for microarray data included in the “limma” library (http://bioconductor.org/packages/release/bioc/html/limma.html) [24]. The accepted cut-off criteria were based on both differences in expression fold change greater than abs. 2 and an adjusted *p*-value ≤ 0.05. Genes that fulfilled the assumed selection criteria were considered to be significantly different and additional analyses were conducted on these genes.

Raw data files and a technical description were also deposited in the Gene Expression Omnibus (GEO) repository at the National Center for Biotechnology Information (http:/www.ncbi.nlm.nih.gov/geo/) under the GEO accession number: GSE106239.

### 4.6. Assignment of Differentially Expressed Genes to Relevant Gene Ontology (GO) Terms

Sets of differentially expressed genes were subjected to functional annotation and clusterization using the DAVID (Database for Annotation, Visualization and Integrated Discovery) web-based bioinformatics tools [25]. Gene symbols for differentially expressed genes were uploaded to DAVID by the “RDAVIDWebService” BioConductor library (https://bioconductor.org/packages/release/bioc/html/RDAVIDWebService.html) [26], where the selection of significantly enriched ontological terms related to the regulation of pluripotency was performed. The results were visualized using the GOplot library [27]. Differentially expressed genes assigned to each GO term were also subjected to a hierarchical clusterization algorithm and presented as heatmaps.

### 4.7. RT-qPCR Evaluation

Real Time-PCR reactions were performed using the PrimePCR^TM^ Custom Plates (Bio-Rad, Hercules, CA, USA) and the specific synthesized primers for each gene: *LIN28A*, *DPPA4*, *PRDM14*, *CER1*, *SFRP2*, *POU5F1*, *SOX2*, *PSMD5*, *SALL4*, *NODAL*, *NANOG*. cDNA samples were analyzed for genes of interest and for the reference gene β-2-microglobulin (B2M). The expression level for each target gene was calculated as −2^ΔΔ*C*t^. The reaction was performed in triplicate for the gene of interest.

## 5. Conclusions

In this paper, we have demonstrated that hiPSCs generated in our laboratory (GPCCi001-A cell line) according to our protocol possess the ability to differentiate into chondrocyte-like cells. The gene expression profile analysis showed that these differentiated cells lost their pluripotency nature. Furthermore, we have demonstrated that our genes involved in self-renewal and pluripotency remained highly expressed in this hiPSC cell line. Therefore, we have created an hiPSC cell line that remains stable during prolonged culture and that maintains the capacity to differentiate into the derivatives of three primary germ layers. Importantly, these chondrocyte-like cells demonstrate a very low tumorigenic potential, which may make these cells ideal for use in future clinical studies.

## Supplementary Materials

Supplementary materials can be found at http://www.mdpi.com/1422-0067/19/2/550/s1.

## Abbreviations

B2M	Β-2 microglobulin
BMP-4	Bone morphogenetic protein 4
ChiPS	Chondrocyte-like cells differentiated from human induced pluripotent stem cells
CER1	Cerbeus, DAN family BMP antagonist
c-Myc	MYC proto-oncogene
DAVID	Database for Annotation, Visualization and Integrated Discovery
DPPA4	Developmental pluripotency associated 4
EBs	Embryoid bodies
ESRG	Embryonic stem cell related (non-protein coding)
FGF-2	Basic fibroblasts growth factor
GEO	Gene Expression Omnibus
GFs	Growth factors
GO	Gene Ontology
GPCCi001-A	Human induced pluripotent stem cell line created in Greater Poland Cancer Centre
hESCs	Human embryonic stem cells
hiPSCs	Human induced pluripotent stem cells
HIST1H3A	Histone cluster 1 H3 family member a
HIST1H3I	Histone cluster 1 H3 family member i
HIST1H2BM	Histone cluster 1 H2B family member m
HOTAIRM1	HOXA transcript antisense RNA, myeloid-specific 1
HOXA	Homeobox A cluster
IGF-1	Insulin-like growth factor 1
KLF4	Kruppel-like factor 4
LIN28A	Lin-28 homolog A
L1TD1	LINE 1 type transposase domain
NANOG	Nanog homeobox
NODAL	Nodal growth differentiation factor
NPC	Neural precursor cells
OCT4	Octamer-binding transcription factor 4
PDGF	Platelet-derived growth factor
POU5F1	POU class 5 homeobox 1
PRDM14	PR/SET domain 14
PWAR5	Prader Willi/Angelman region RNA 5
PWARSN	Prader Willi/Angelman region RNA, SNRPN neighbor
REX1	Ring-exported protein 1
RMA	Robust Multiarray Average
RNU5B-1	RNA, U5B small nuclear 1
RNA5SP221	RNA, 5S ribosomal pseudogene 221
SALL4	Spalt like transcription factor 4
SCARNA1	Small Cajal body-specific RNA 1
SCARNA8	Small Cajal body-specific RNA 8
SCARNA23	Small Cajal body-specific RNA 23
SCs	Stem Cells
SFRP2	Secreted frizzled related protein 2
SNORA14B	Small nucleolar RNA, H/ACA box 14B
SNORD116-21	Small nucleolar RNA, C/D box 116-21
SNORD116-1	Small nucleolar RNA, C/D box 116-1
SNORD116-30	Small nucleolar RNA, C/D box 116-30
SOX2	SRY (Sex determining region Y)-box 2
SOX6	SRY (Sex determining region Y)-box 6
SOX9	SRY (Sex determining region Y)-box 9
SSEA4	Stage-specific embryonic antigen-4
TGF-β3	Transforming growth factor β 3
Wnt	Wingless-type MMTV integration site family
