# Supplementary material for: Expression of Pluripotency Genes in Chondrocyte-Like Cells Differentiated from Human Induced Pluripotent Stem Cells
Source: Int J Mol Sci. 2018 Feb 12;19(2):550. doi: 10.3390/ijms19020550 (PMC5855772; doi:10.3390/ijms19020550)
Supplement: Supplementary file 1 [file ijms-19-00550-s001.docx]

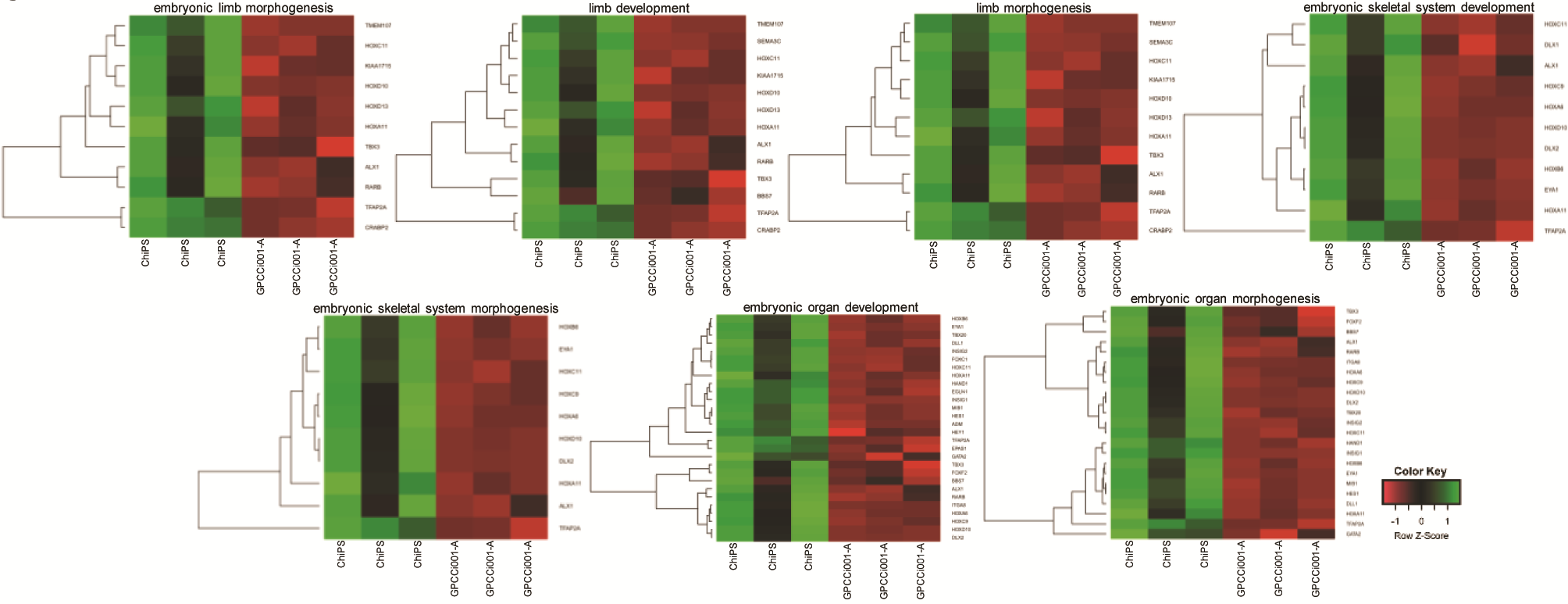


**Figure S1.** Heatmap graphs of the genes in the experimental groups: ChiPS vs. GPCCi001-A from the following GO terms engaged in stages of chondrogenesis: “embryonic limb morphogenesis”, “limb development”, “limb morphogenesis”, “embryonic limb morphogenesis”, “embryonic skeletal system development”, “embryonic skeletal system morphogenesis”, “embryonic organ development”, and “embryonic organ morphogenesis”. Arbitrary signal intensity acquired from the microarray analysis is represented by the colours (green = higher expression; red = lower expression). Log2 signal intensity values for any single gene were resized to row Z-score scales. Genes belonging to the relevant GO term are described by their symbols.


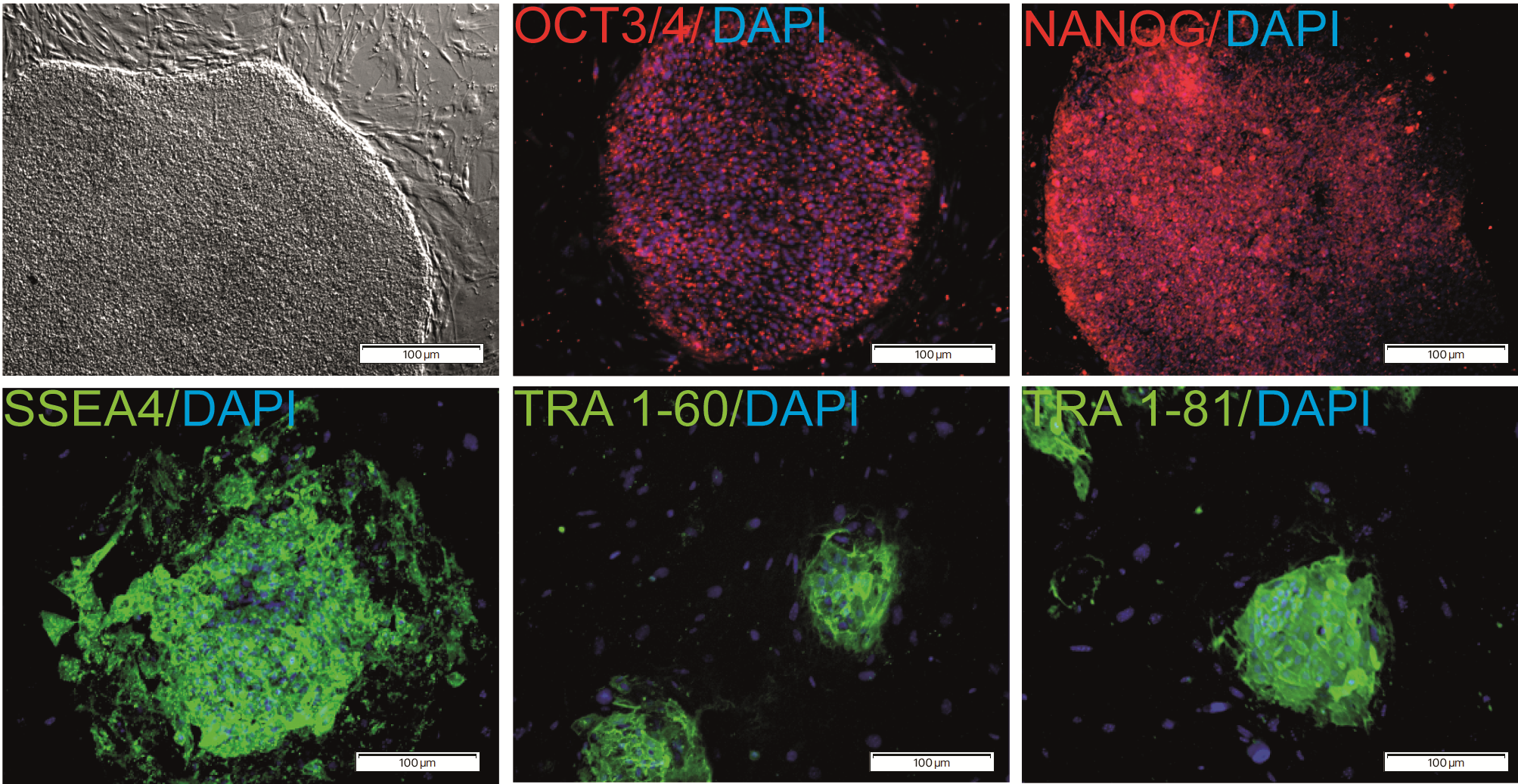


**Figure S2.** GPCCi001-A cell line reprogrammed from primary human dermal fibroblasts reveals pluripotency nature during pro-longed culture (after reprograming process hiPSCS underwent chondrogenic differentiation or they were further cultured for microarray study as positive control and for immunofluorscene analysis). These hiPSCs demonstrate the presence of intra-(OCT3/4 and NANOG) and extracellular (SSEA4, TRA-1-60 and TRA-1-81), desirable proteins.
